# Risk factors for developing a cutaneous injection-related infection among injection drug users: a cohort study

**DOI:** 10.1186/1471-2458-8-405

**Published:** 2008-12-09

**Authors:** Elisa Lloyd-Smith, Evan Wood, Ruth Zhang, Mark W Tyndall, Julio SG Montaner, Thomas Kerr

**Affiliations:** 1 BC Centre for Excellence in HIV/AIDS, St. Paul's Hospital 608-1081 Burrard Street, Vancouver, BC V6Z 1Y6, Canada; 2 Department of Health Care and Epidemiology, University of British Columbia, Vancouver, Canada; 3 Department of Medicine, University of British Columbia, Vancouver, Canada

## Abstract

**Background:**

Cutaneous injection-related infections (CIRI), such as abscesses and cellulitis, are common and preventable among injection drug users (IDU). However, risk factors for CIRI have not been well described in the literature. We sought to characterize the risk factors for current CIRI among individuals who use North America's first supervised injection facility (SIF).

**Methods:**

A longitudinal analysis of factors associated with developing a CIRI among participants enrolled in the Scientific Evaluation of Supervised Injecting (SEOSI) cohort between January 1, 2004 and December 31, 2005 was conducted using generalized linear mixed-effects modelling.

**Results:**

In total, 1065 participants were eligible for this study. The proportion of participants with a CIRI remained under 10% during the study period. In a multivariate generalized linear mixed-effects model, female sex (Adjusted Odds Ratio (AOR) = 1.68 [95% Confidence Interval (CI): 1.16–2.43]), unstable housing (AOR = 1.49 [95% CI: 1.10–2.03]), borrowing a used syringe (AOR = 1.60 [95% CI: 1.03–2.48]), requiring help injecting (AOR = 1.42 [95% CI: 1.03–1.94]), and injecting cocaine daily (AOR = 1.41 [95% CI: 1.02–1.95]) were associated with an increased risk of having a CIRI.

**Conclusion:**

CIRI were common among a subset of IDU in this study, including females, those injecting cocaine daily, living in unstable housing, requiring help injecting or borrowing syringes. In order to reduce the burden of morbidity associated with CIRI, targeted interventions that address a range of factors, including social and environmental conditions, are needed.

## Background

Injection drug use remains a major public health concern worldwide. While researchers and policy makers have focused much attention on the transmission of blood-borne viruses (e.g., human immunodeficiency virus [HIV], hepatitis C virus [HCV]) among injection drug users (IDU) [1-3], considerably less attention has been devoted to the problem of bacterial infections of the skin [4]. A recent report by the United States Centres for Disease Control and Prevention highlighted the dearth of research on cutaneous injection-related infections (CIRI) among IDU and cited reports of recent and dramatic increases in CIRI, including abscesses and cellulitis, among IDU in England [5]. In addition, CIRI are the primary reason that IDU seek treatment at an emergency department in some settings [6].

The prevalence of CIRI among IDU typically ranges from 10% to 30% [7,8]. The variability in prevalence estimates may be due to differences in measurement of the occurrence of CIRI (e.g., reporting current vs. ever having a CIRI), the definition of CIRI (e.g., injection-related vs. any drug-related) and the fact that there are currently no standard guidelines with regard to reporting of CIRI based on severity [4]. Other factors may be the exploration of different risk factors for developing CIRI across settings, for example differences in types of drugs used (e.g., black tar heroin in California, United States vs. white heroin in British Columbia, Canada), intensity of drug use, and availability and access to clean injection paraphernalia [9].

However, certain factors have been shown to be consistently associated with developing a CIRI in particular cities. For example: injecting under the skin (also known as "skin popping") in San Francisco and Glasgow [8,10,11]; frequent injection in Amsterdam and Vancouver [12,13]; and injection of heroin plus cocaine (i.e. "speedballs") in San Francisco and Amsterdam [8,13] have been associated with CIRI. However, most of these analyses were based on cross-sectional investigations few prospective studies exist. We conducted the present longitudinal study to characterize risk factors of developing a CIRI among IDU.

## Methods

### Data source: Scientific Evaluation of Supervised Injection

In September 2003, a comprehensive evaluation of Insite, North America's first supervised injection facility (SIF), was initiated. Users of the SIF, located in Vancouver's Downtown Eastside (DTES) neighbourhood, were randomly invited to enroll in a prospective cohort study known as the Scientific Evaluation of Supervised Injection (SEOSI) and have since been interviewed semi-annually [14]. The methodological details have been described elsewhere [15]. Briefly, to be recruited into the SEOSI cohort, individuals had to have performed at least two injections at the SIF, been at least 19 years old and provided informed consent. Furthermore, the questionnaire is interviewer-administered and elicits a range of information, including information specific to socio-demographic characteristics, risk behaviours, and involvement in addiction treatment. This is followed by blood testing for HIV and HCV for those who previously tested negative and a nurse-administered questionnaire on health status. A database in the SIF tracks key events, including utilization and the types of drug being injected. The SEOSI cohort has received approval from the Providence Health Care/University of British Columbia Ethics Board.

To be eligible for this study, participants must have completed both the interviewer-administered and nurse-administered baseline questionnaires during the study period (January 1, 2004 to December 31, 2005.)

### Outcome Measure and Explanatory Variables

The primary outcome for this analysis (dependent variable) was a current CIRI reported to and visually confirmed (e.g., pain, redness, induration, and fluctuation) by the study nurse in response to the question "Do you presently have any sores or abscesses from where you have been injecting?". Explanatory variables examined in this analysis included: age (per year older); sex (female vs. male); unstable housing, defined as living in a single room occupancy hotel, shelter, recovery or transition house, jail, on the street, or having no fixed address as opposed to living in an apartment or house (yes vs. no); residence in the DTES (yes vs. no); sex trade involvement (yes vs. no); borrowing used syringes (yes vs. no); requiring help injecting (yes vs. no); using puddle water for injecting (yes vs. no); injecting cocaine daily (yes vs. no); injecting heroin daily (yes vs. no), injecting crack cocaine daily (yes vs. no); injecting crystal methamphetamine daily (yes vs. no); injecting "speedballs" daily (yes vs. no); the proportion of all injections at SIF (always vs. < always); HIV serostatus (positive vs. negative); and HCV serostatus (positive vs. negative). Variable definitions were consistent with previous work [16-19]. All variables referred to the six months prior to the interview, except for unstable housing and residence in the DTES, which referred to resident status at the more recent interview.

### Statistical Analysis

The proportion of SEOSI participants who reported having a CIRI was inspected graphically over time. Univariate and multivariate statistics, including generalized linear mixed-effects modeling, were used to examine factors associated with having a CIRI over time. Generalized linear mixed-effects modeling is a longitudinal technique that analyzes individual trajectories and produces correlates. This analytic technique was chosen because of its flexibility in variable parameters (e.g., fixed, time-updated, random), its ability to capture heterogeneity of subjects and within-subject correlation, and its attempt to identify individual-level factors [20]. Independent variables were either fixed (e.g., sex) or time-updated (e.g., age, all behavioural variables considered, HIV and HCV serostatus) in this model. Random variation between individuals was accounted for by using random intercepts. Variables significant at the univariate level (*p *< 0.05) were included in the multivariate model. The data were analyzed using SAS version 9.1 (SAS Institute, Cary, NC, USA.) All reported *p*-values were two-tailed.

## Results

**Figure 1:**
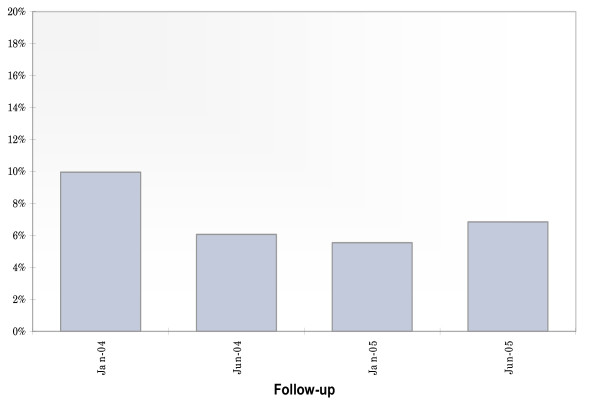
**Proportion of SEOSI participants reporting a CIRI (January 1, 2004 – December 31, 2005, n = 1065)**.

Of the 1090 participants recruited into the SEOSI cohort since November 2003, 1065 (97%) completed both a baseline interviewer-administered questionnaire and a nurse-administered questionnaire after recruitment into SEOSI cohort. Among these participants, 877 (82%) returned for at least one follow-up visit, and 312 (29%) were female. The median age was marginally younger for those who reported a CIRI at baseline when compared with those who did not at baseline (36 [IQR: 31–43] vs. 39 [IQR: 33–45], *p *= 0.095). As shown in Figure 1, the proportion of participants reporting a current CIRI in this study was fairly consistent over the two year study period, ranging from 6% to 10%, although the proportion declined slightly between the baseline and first follow-up visit. At baseline, 106 (10%) of participants reported a CIRI. There were 14 (1%) individuals with missing data on HIV serostatus and 33 (3%) individuals with missing data on HCV serostatus at baseline; due to these small counts, these individuals were excluded from further analyses.

Using longitudinal methods, factors associated with reporting a current CIRI at the univariate and multivariate level are presented in Table 1. In univariate analyses, being older and reporting use of the SIF for all injections was associated with a decreased likelihood of developing a current CIRI. Female sex, living in unstable housing, involvement in the sex trade, borrowing a used syringe, requiring help injecting, injecting cocaine daily, injecting heroin daily, and injecting "speedballs" daily were positively associated with reporting a current CIRI.

**Table 1:** Univariate and multivariate analysis of developing a CIRI among SEOSI participants (n = 1065)

**Variable**	**OR**	**(95% - CI)**	**AOR**	**(95% - CI)**
Age				
(per year older)	0.98	(0.96 – 1.00)	1.00	(0.98 – 1.02)
Sex				
(Female vs. Male)	1.90	(1.39 – 2.58)	1.68	(1.16 – 2.43)
Unstable housing				
(Yes vs. No)	1.56	(1.15 – 2.12)	1.49	(1.10 – 2.03)
DTES residence				
(Yes vs. No)	1.33	(0.96 – 1.85)		
Sex trade*				
(Yes vs. No)	1.74	(1.24 – 2.45)	1.02	(0.67 – 1.56)
Borrowing syringes*				
(Yes vs. No)	1.88	(1.22 – 2.88)	1.60	(1.03 – 2.48)
Requiring help inject*				
(Yes vs. No)	1.85	(1.37 – 2.50)	1.42	(1.03 – 1.94)
Use puddle to inject*				
(Yes vs. No)	1.32	(0.83 – 2.11)		
Cocaine injection*				
(Daily vs. Not)	1.66	(1.23 – 2.25)	1.41	(1.02 – 1.95)
Heroin injection*				
(Daily vs. Not)	1.53	(1.14 – 2.04)	1.26	(0.93 – 1.72)
Crack injection*				
(Daily vs. Not)	1.54	(0.96 – 2.46)		
Crystal meth. injection*				
(Daily vs. Not)	1.48	(0.73 – 3.02)		
Speedball injection*				
(Daily vs. Not)	2.00	(1.35 – 2.96)	1.37	(0.89 – 2.11)
SIF use				
(Always vs. Not)	0.47	(0.23 – 0.94)	0.58	(0.29 – 1.19)
HIV serostatus				
(Yes vs. No)	1.23	(0.85–1.77)		
HCV serostatus				
(Yes vs. No)	1.35	(0.81 – 2.25)		

Note: *activity past 6 months, DTES = downtown eastside, meth. = methamphetamine, SIF = supervised injection facility

As displayed in the multivariate model in Table 1, participants who reported a current CIRI were more likely to be female (Adjusted OR (AOR) = 1.68 [95% Confidence Intervals {CI}: 1.16–2.43]); live in unstable housing (AOR = 1.49 [95% CI: 1.10–2.03]); borrow used syringes (AOR = 1.60 [95% CI: 1.03–2.48]); require help injecting (AOR = 1.42 [95% CI: 1.03–1.94]); and inject cocaine daily (AOR = 1.41 [95% CI: 1.02–1.95]).

## Discussion

In this study we found that the proportion of IDU reporting a CIRI remained within the range of six to 10 per cent over a median follow-up of 12.6 (IQR: 6.2–17.7) months after SIF recruitment. The level of CIRI is relatively low in the context of previously reported prevalence (10–30% [7,8]). However, considering that it is based on reporting a current infection, the level in this study is concerning. Furthermore, our results indicate that being female, living in unstable housing, borrowing syringes, requiring help injecting, and injecting cocaine daily were independently associated with developing a CIRI.

The observed associations between female sex, daily cocaine injection, living in unstable housing and an elevated risk of having a CIRI are congruent with previous analyses. The link between being female and having a CIRI echoes the findings of previous studies [10,12,13], and may reflect, in part, the complex gender dynamics that exist within injection drug using populations where women are often dependent on men for the attainment and administration of drugs [21].

With regard to the association between cocaine injection and development of CIRI [12,13], cocaine's anaesthetic properties may make it more difficult for individuals to know whether or not they are hitting a vein (as opposed to injecting in the surrounding tissue or skin), resulting in trauma through repetitive attempts to access the vein [22,23]. Missing a vein increases vulnerability for CIRI since injecting into the surrounding tissue creates a niche environment in which bacteria can thrive [9]. Further, due to cocaine's short half-life in comparison to heroin, it is often injected many more times than heroin, which also increases the likelihood of CIRI and transmission of blood-borne viruses such as HIV [16]. Indeed, as indicated by our findings and others, intensity of drug use appears to play a role in CIRI development, as individuals who inject at least once daily have been repeatedly identified to be at elevated risk for developing a CIRI [12,13].

The association between homelessness and an injection site infection has been reported [24]. According to the 'risk environment' framework, as proposed by Rhodes et al., structural and environmental factors are important to consider when assessing risks for drug-related harms as they shape the context in which individual behaviour occurs [24]. It may be that those in our study who reported living in unstable housing may also frequent risky injecting environments, which in turn lead to rushed injections (i.e., not taking time to go through every step of the injection process to ensure a safer injection) or injecting in a high-risk location like the groin for a 'quick fix' [25]. A recent review of homelessness found that between 15–50% of homeless individuals inject drugs, and it was further reported that breaks of the skin were common among such individuals, often leading to bacterial infections due to a lack of hygiene [26]. In addition, the small size, shared facilities and often unhygienic environment of single room occupancy hotels that are common in the DTES promote disease transmission [27].

Among the novel findings in the present study are the associations between CIRI development and borrowing syringes and requiring help injecting. Borrowing used syringes is known to be a strong risk factor for blood-borne viral transmission [28,29]. Our study shows that the transmission of CIRI-related bacteria via sharing of syringes should also be considered by IDU, health professionals, and public health practitioners. However, it is also possible that sharing syringes is not the active vector in this transmission and that this transmission is by other injection drug paraphernalia. Requiring help injecting, a risk factor for CIRI in this study, may increase risk of exposure to bacteria when the individual who is administering the injection injects themselves before injecting the person who requires assistance (i.e., "second on the needle").

This study has several limitations. Firstly, we were unable to examine "skin popping" as an independent variable in this study due to a low number of participants reporting this behaviour. This may be due to the fact that the practice is more commonly associated with injection of "black tar" heroin, a type rarely used in Vancouver. Given that our "skin popping" question in the study questionnaire pertained to intentional "skin popping" it is also possible that participants who injected subcutaneously or intramuscularly by mistake were not captured. Secondly, our study relies on self-report and therefore is potentially vulnerable to social desirability bias. However, we know of no reason to suspect differential reporting between participants with or without CIRI. Thirdly, it is possible that individuals who inject at the SIF are different from those who do not. A study by Wood et al. found that IDU that used the SIF were more likely to be at a higher risk of blood-borne disease infection and overdose compared with IDU who did not use the SIF [30]. Therefore, our results may not be generalizable to the broader local IDU population. However, the SEOSI cohort was randomly recruited from within the SIF [15]. Therefore, we believe that our sample is representative of SIF users. Fourthly, the external validity of this study should be interpreted with caution, as Vancouver's DTES neighbourhood is unique due to its large open drug scene and the high prevalence of cocaine injection. Finally, this study investigates only CIRI related to injection drug use and not other behaviours for example. However, we feel this is an important distinction as it serves to reduce misclassification bias based on reporting CIRI that may be related to other factors such as picking the skin induced by cocaine psychosis [16].

The prevalence of CIRI among IDU in this study suggests that a higher priority should be placed on reducing the incidence of these preventable infections. Since a positive impact of the SIF on access to assessment, care, and treatment of CIRI has been noted [31], it is likely that the rate of CIRI observed here may be lower than the rate observed in the broader community. Combining harm reduction (e.g., needle exchange programs and supervised injection facilities) and treatment services may be of value to prevent and/or reduce the risk for CIRI development. Specifically, integrating wound management care into existing harm reduction services, such as needle exchange programs and SIF, in community settings has been found to be feasible, cost-effective and beneficial for preventing and treating CIRI and other related skin infections such as necrotizing fasciitis [32]. Expansion of such programs among harm reduction services may be reasonable, especially as many IDU remain medically underserved [33].

## Conclusion

In summary, we found that over a two-year period that between six and 10 percent of IDU presented with a CIRI. Risk factors for CIRI development included being female, living in unstable housing, borrowing used syringes, requiring help injecting and injecting cocaine daily. These findings collectively point to the need to develop a range of interventions that target the various individual, social and environmental risks for CIRI development.

## Competing interests

The authors declare that they have no competing interests.

## Authors' contributions

ELS conceived and designed the study and drafted the manuscript. ELS and RZ performed the statistical analyses. ELS, TK, RH, EW, MT, contributed to the design and coordination of the study and provided assistance with interpretation of the results and the draft of the manuscript. All authors read and approved the final manuscript.

## Pre-publication history

The pre-publication history for this paper can be accessed here:



## References

[B1] Alter MJ (1997). Epidemiology of Hepatitis C. Hepatology.

[B2] Holmberg SD (1996). The estimated prevalence and incidence of HIV in 96 large US metropolitan areas. Am J Public Health.

[B3] Hagan H, Des Jarlais DC (2000). HIV and HCV infection among injecting drug users. Mt Sinai J Med.

[B4] del Giudice P (2004). Cutaneous complications of intravenous drug abuse. Br J Dermatology.

[B5] Irish C, Maxwell R, Dancox M, Brown P, Trotter C, Verne J (2007). Skin and soft tissue infections and vascular disease among drug users, England. Emerg Infect Dis.

[B6] Palepu A, Tyndall MW, Leon H, Muller J, O'Shaughnessy MV, Schechter MT (2001). Hospital utilization and costs in a cohort of injection drug users. CMAJ.

[B7] Binswanger IA, Kral AH, Bluthenthal RN, Rybold DJ, Edlin BR (2000). High prevalence of abscesses and cellulitis among community-recruited injection drug users in San Francisco. Clin Infect Dis.

[B8] Murphy EL, DeVita D, Liu H, Vittinghoff E, Leung P, Ciccarone DH (2001). Risk factors for skin and soft-tissue abscesses among injection drug users: a case-control study. Clin Infect Dis.

[B9] Ciccarone D, Bourgois P (2003). Explaining the geographical variation of HIV among injection drug users in the United States. Subst Use Misuse.

[B10] Taylor A, Hutchinson S, Lingappa J, Wadd S, Ahmed S, Gruer L (2005). Severe illness and death among injecting drug users in Scotland: a case-control study. Epidemiol Infect.

[B11] CDC (2001). Soft tissue infections among injection drug users–San Francisco, California, 1996–2000. MMWR Morb Mortal Wkly Rep.

[B12] Lloyd-Smith E, Kerr T, Hogg RS, Li K, Montaner JSG, Wood E (2005). Prevalence and correlates of abscesses among a cohort of injection drug users. Harm Reduct J.

[B13] Spijkerman IJ, van Ameijden EJ, Mientjes GH, Coutinho RA, Hoek A van den (1996). Human immunodeficiency virus infection and other risk factors for skin abscesses and endocarditis among injection drug users. J Clin Epidemiol.

[B14] Tyndall M, Kerr T, Zhang R, King E, Montaner JG, Wood E (2005). Attendance, drug use patterns, and referrals made from North America's first supervised injection facility. Drug Alcohol Depend.

[B15] Wood E, Kerr T, Lloyd-Smith E, Buchner C, Marsh DC, Montaner JSG (2004). Methodology for evaluating Insite: Canada's first medically supervised safer injection facility for injection drug users. Harm Reduct J.

[B16] Tyndall MW, Currie S, Spittal P, Li K, Wood E, O'Shaughnessy MV (2003). Intensive injection cocaine use as the primary risk factor in the Vancouver HIV-1 epidemic. AIDS.

[B17] Strathdee SA, Patrick DM, Currie SL, Cornelisse PG, Rekart ML, Montaner JS (1997). Needle exchange is not enough: lessons from the Vancouver injecting drug use study. AIDS.

[B18] Miller CL, Johnston C, Spittal PM, Li K, Laliberte N, Montaner JS (2002). Opportunities for prevention: Hepatitis C prevalence and incidence in a cohort of young injection drug users. Hepatology.

[B19] Craib KJ, Spittal PM, Wood E, Laliberte N, Hogg RS, Li K (2003). Risk factors for elevated HIV incidence among Aboriginal injection drug users in Vancouver. CMAJ.

[B20] Fitzmaurcie GM, Laird NM, JH W (2004). Applied Longitudinal Analysis.

[B21] Bourgois P, Prince B, Moss A (2004). The everyday violence of Hepatitis C among young women who inject drugs in San Francisco. Human Organization.

[B22] (2003). Sydney Medically Supervised Injecting Centre Final report of the evaluation of the Sydney Medically Supervised Injecting Centre.

[B23] Rhodes T, Briggs D, Kimber J, Jones S, Holloway G (2007). Crack-heroin speedball injection and its implications for vein care: qualitative study. Addiction.

[B24] Rhodes T (2002). The 'risk environment': a framework for understanding and reducing drug-related harm. International J Drug Policy.

[B25] Rhodes T, Stoneman A, Hope V, Hunt N, Martin A, Judd A (2006). Groin injection in the context of crack cocaine and homelessness: from 'risk boundary' to 'acceptable risk'?. International J Drug Policy.

[B26] Raoult D, Foucault C, Brouqui P (2001). Infections in the homeless. Lancet Infect Dis.

[B27] Shannon K, Ishida T, Lai C, Tyndall MW (2006). The impact of unregulated single room occupancy hotels on the health status of illicit drug users in Vancouver. International J Drug Policy.

[B28] Patrick DM, Strathdee SA, Archibald CP, Ofner M, Craib KJ, Cornelisse PG (1997). Determinants of HIV seroconversion in injection drug users during a period of rising prevalence in Vancouver. International Journal of STD & AIDS.

[B29] Strathdee SA, Patrick DM, Archibald CP, Ofner M, Cornelisse PG, Rekart M (1997). Social determinants predict needle-sharing behaviour among injection drug users in Vancouver, Canada. Addiction.

[B30] Wood E, Tyndall M, Li K, Lloyd-Smith E, Small W, Montaner J (2005). Do Supervised Injecting Facilities Attract Higher-Risk Injection Drug Users?. Am J Prev Med.

[B31] Small W, Wood E, Lloyd-Smith E, Tyndall M, Kerr T (2008). Accessing care for injection-related infections through a medically supervised injecting facility: a qualitative study. Drug Alcohol Depend.

[B32] Grau LE, Arevalo S, Catchpool C, R H (2002). Expanding harm reduction services through a wound and abscess clinic. Am J Public Health.

[B33] Young DM, Harris HW, Charlebois ED, Chambers H, Campbell A, Perdreau-Remington F (2004). An epidemic of methicillin-resistant staphylococcus aureus soft tissue infections among medically underserved patients. Arch Surg.

